# In Silico Electrophysiological Investigation of Transient Receptor Potential Melastatin-4 Ion Channel Biophysics to Study Detrusor Overactivity

**DOI:** 10.3390/ijms25136875

**Published:** 2024-06-22

**Authors:** Chitaranjan Mahapatra, Ravindra Thakkar

**Affiliations:** 1 Cardiovascular Research Institute, University of California San Francisco, San Francisco, CA 94158, USA; 2 Paris Saclay Institute of Neuroscience, 91440 Saclay, France; 3 California Institute for Quantitative Biosciences, University of California Berkeley, Berkeley, CA 94720, USA

**Keywords:** urinary incontinence, TRPM4 ion channel, action potential, computational modeling

## Abstract

Enhanced electrical activity in detrusor smooth muscle (DSM) cells is a key factor in detrusor overactivity which causes overactive bladder pathological disorders. Transient receptor potential melastatin-4 (TRPM4) channels, which are calcium-activated cation channels, play a role in regulating DSM electrical activities. These channels likely contribute to depolarizing the DSM cell membrane, leading to bladder overactivity. Our research focuses on understanding TRPM4 channel function in the DSM cells of mice, using computational modeling. We aimed to create a detailed computational model of the TRPM4 channel based on existing electrophysiological data. We employed a modified Hodgkin-Huxley model with an incorporated TRP-like current to simulate action potential firing in response to current and synaptic stimulus inputs. Validation against experimental data showed close agreement with our simulations. Our model is the first to analyze the TRPM4 channel’s role in DSM electrical activity, potentially revealing insights into bladder overactivity. In conclusion, TRPM4 channels are pivotal in regulating human DSM function, and TRPM4 channel inhibitors could be promising targets for treating overactive bladder.

## 1. Introduction

People encounter diverse health issues that influence their well-being. Experiencing a non-lethal heart attack can significantly alter one’s perspective on life, prompting a heightened awareness of blood pressure, dietary modifications, the adoption of exercise regimens, decreased smoking, and various lifestyle adjustments. The gravity of a health ailment might not always be obvious but can profoundly affect one’s quality of life. Many of us may overlook the simplicity of typical urinary bladder function. The primary physiological roles of a healthy urinary bladder are to store urine and facilitate voluntary micturition (the act of urinating) [1,2]. However, overactive bladder syndrome (OAB, also known as overactive detrusor function) presents symptoms such as urgency for urination with or without urge incontinence, frequent urination, and nocturia (waking at night to urinate) [3,4,5,6,7,8]. The impact of OAB on an individual’s quality of life, on the healthcare system, and on the workforce carries considerable economic burdens [9,10,11]. The exact cause of OAB remains largely unknown. The detrusor smooth muscle (DSM) within the bladder wall plays a crucial role in bladder function [12,13]. During urine storage, DSM cells relax, allowing the bladder to hold urine for extended periods without leakage. When urination is necessary, DSM cells contract synchronously with the coordinated action of internal and external urethral sphincters [14,15]. This coordinated activity, orchestrated by the brain and spinal cord, triggers DSM contraction for micturition. In OAB, involuntary contractions of DSM cells can occur during urine storage, leading to leakage [16,17]. Lifestyle changes and pharmacological therapy are among the strategies available to manage OAB and mitigate its complications [18,19]. Pharmacological therapy devoid of side effects is crucial in pathological situations to ensure optimal patient outcomes and minimize treatment-associated risks [20]. It allows for effective management of symptoms while preserving patient safety and quality of life. Treatment options for OAB often involve medications like anticholinergics and beta-3 adrenergic agonists, designed to alleviate symptoms by calming the DSM and lessening the urge to urinate frequently [21,22]. Nonetheless, these medications can lead to side effects such as dry mouth, constipation, blurred vision, and cognitive decline with anticholinergics, as well as potential hypertension with beta-3 adrenergic agonists [23,24,25]. Therefore, understanding the DSM electrophysiology becomes essential to explore new pharmacological targets to mitigate the side effects induced by conventional medications.

Several experimental studies have revealed that DSM cells, found in various species, including humans, exhibit enhanced spontaneous phasic contractions, contributing to the development of an overactive bladder [26,27]. The initiation of spontaneous phasic contractions is triggered by the generation of evoked spontaneous action potentials (APs) and accompanying calcium (Ca^2+^) dynamics [28,29,30,31]. A thorough biophysical explanation of DSM AP generation is crucial for comprehending spontaneous contractions and for exploring novel pharmacological targets for overactive bladder treatment. The electrical activities in all excitable tissues are regulated by the influx and efflux of charged ions through a network of ion channels situated at the cell membrane. In a resting state, electrically excitable cells maintain a specific resting membrane potential, which varies among different tissue types, including neuronal cells, cardiac cells, and smooth muscle cells. A transient rise in cytoplasmic calcium [Ca^2+^]_i_ is an important reason behind DSM cell contraction [32,33]. The influx of extracellular Ca^2+^ ions occurs mainly via voltage-dependent L-type and T-type Ca^2+^ channels [34,35,36]. The sarco/endoplasmic reticulum (SR) is the principal Ca^2+^ store participating in the initial rapid increase in [Ca^2+^]_i_ by supplying Ca^2+^ via Ca^2+^ release mechanism by SR [37,38,39]. T-type Ca^2+^ channels are activated to depolarize the membrane potential until reaching the threshold potential, which then triggers the opening of L-type Ca^2+^ channels. Ca^2+^ influx via the L-type Ca^2+^ channel is essential for the rising phase of the DSM action potential, whereas various potassium (K^+^) channels mediate the repolarization and, afterwards, a hyper-polarization period of the action potential, respectively [34,40,41,42]. Of the large family of mammalian K^+^ channels, the Ca^2+^-dependent K^+^ channel, voltage-dependent K^+^ channel, and the delayed rectifier K^+^ channel are incorporated along with L-type and T-type Ca^2+^ channels in AP generation [41,42,43,44]. In addition, the Ca^2+^-dependent K^+^ channels in DSM cells are categorized into large (BK), intermediate (IK), and small (SK) conductance ion channels [45,46,47,48]. Several research groups have recently conducted an intriguing study revealing a novel regulatory mechanism of the transient receptor potential melastatin 4 (TRPM4) ion channel in modulating DSM cell excitability [49,50]. The TRPM4 channel has been shown to play a crucial role in regulating the resting membrane potential and basal tone of various smooth muscle cells. Through the utilization of the whole-cell voltage clamp method, researchers have successfully recorded TRPM4 channel currents in single smooth muscle cells within urinary bladder tissues from diverse animal subjects [49,51,52,53,54,55,56]. Their findings suggest that TRPM4 could serve as a novel therapeutic target for alleviating symptoms of OAB.

**Figure 1:**
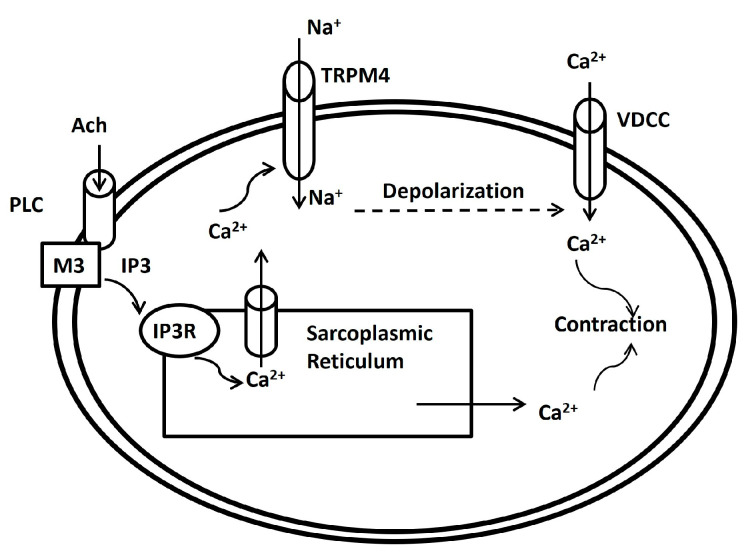
Schematic illustration of the proposed physiological role for TRPM4 channels in DSM cells. According to the postulated mechanism, the TRPM4 channels via sarcoplasmic reticulum Ca^2+^-dependent activation participate in a positive feedback loop to maximize DSM contractility by providing Na^+^-depolarizing conductance.

TRPM4 functions as a Ca^2+^-activated non-selective cation channel, inducing cell membrane depolarization via sodium (Na^+^) or K^+^ entry and subsequent activation of L-type Ca^2+^ channels (Figure 1) [54]. In one study, the application of 9-phenanthrol resulted in hyperpolarization of DSM cell membranes, indicating the first evidence of TRPM4 regulation of human DSM cell resting membrane potential [55]. Moving forward, the researchers conducted in vitro experiments to investigate the impact of TRPM4 on spontaneous and phasic contractions of human DSM isolated strips. Inhibition of TRPM4 channels with 9-phenanthrol significantly reduced spontaneous and phasic contraction amplitude, muscle force integrality, contraction duration, contraction frequency, and muscle tone of the DSM, highlighting TRPM4′s critical role in human DSM modulation under physiological conditions. The illustration in Figure 1 explains the possible associated TRPM4 ion channel activation mechanism in DSM cells. Acetylcholine (Ach), a muscarinic agent, activates the muscarinic receptors (M3) at the membrane [57,58,59]. Then, phospholipase C (PLC) is activated, leading to the hydrolysis of phosphatidylinositol 4,5-bisphosphate (PIP2) into inositol trisphosphate (IP3) and diacylglycerol (DAG). IP3 then binds to IP3 receptors on the sarcoplasmic reticulum (SR), causing the release of Ca^2+^ ions from the SR stores into the cytoplasm [53,60]. One portion of the elevated [Ca^2+^]_i_ activates the TRPM4 ion channel and allows the influx of Na^+^ ions (or K^+^ ions) to depolarize the membrane. When the depolarization crosses the threshold potential, the L-type Ca^2+^ channel, which is a voltage-dependent calcium channel (VDCC), opens and allows an influx of extracellular Ca^2+^ into the cytoplasm. The cytoplasmic Ca^2+^, released from the SR store and via the L-type Ca^2+^ channel, triggers contraction after the generation of the action potential.

Unfortunately, the quantitative description of the modulatory effects of the TRPM4 ion channel on DSM cell electrical properties has not been thoroughly explored due to the highly complex nature of the experimental procedures involved in DSM cell electrophysiology. Unlike cardiac and neuronal electrophysiological studies, obtaining comprehensive electrical recordings from smooth muscle cells is challenging. Consequently, most research focuses on the TRPM4 channel’s impact on DSM contraction, rather than on its effect on passive or active electrical properties, like spike/AP modulation. Understanding the biophysical parameters governing DSM cell electrophysiology is crucial for drug design accuracy. Additionally, there is debate over variations in inward and outward currents in DSM electrophysiology. The redundancy of ion channel types regulates cellular functions and maintains physiological balance, ensuring resilience against perturbations [61]. However, investigations of the redundancy and resilience of TRPM4 concerning other inward current ion channels in DSM cells remain unexplored.

Over the past few decades, computational and mathematical modeling techniques have enhanced our comprehension of intricate biological processes. By simulating diverse scenarios and predicting outcomes, these methods offer insights that may be challenging to attain through traditional experimental approaches [62]. The primary goal of computational biophysical modeling is to provide insights into the structure, function, and dynamics of biological systems that may be difficult or impossible to obtain through experimental methods alone. In the realm of smooth muscle electrophysiology, computational simulations have been instrumental in exploring cellular biophysics and in modulating cellular electrical activities. These models shed light on ion channel function, encompassing aspects such as conductance, ion selectivity, and channel opening, which hold significance in pathological conditions. Moreover, biophysical modeling directly simulates ion flux through membrane channels, facilitating a deeper understanding of their behavior and their involvement in disease processes. While computational models for various types of smooth muscle cells, such as intestinal [63], uterine [64,65,66], ureter [67,68,69], jejunal [70], vas deferens [71,72,73,74], gastric [75,76], mesenteric [77], small bowel [78], urethra [79,80], and arterial [81] smooth muscle cells, have been developed, models for DSM cells are relatively underdeveloped. The models published on DSM electrophysiology have yet to explore the modulatory properties of TRPM4 ion channels in DSP action potentials [82,83,84,85,86,87,88]. The present in silico model aims to elucidate the biophysical mechanisms underlying DSM electrophysiology, and to investigate the impact of the TRPM4 ion channel on the firing rate of DSM action potentials. Our objectives in developing this model are threefold. Firstly, leveraging experimental data, we will simulate and validate the kinetics of TRPM4 ion channels in response to changes in intracellular Ca^2+^ concentration. Secondly, we will integrate these ion channels into a single-compartment biophysical DSM model to simulate APs and explore alterations in AP parameters associated with TRPM4 ion channel activation. Lastly, we seek to derive novel biological insights to corroborate existing hypotheses drawn from various experiments, and propose new hypotheses for future research endeavors.

## 2. Results

**Figure 2:**
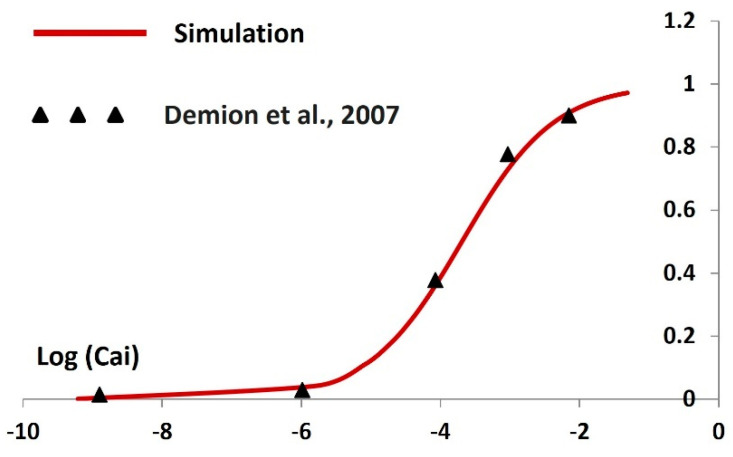
Simulated steady-state activation curve showing log (Cai). The solid line represents the result from simulation, where the solid filled triangle shows the adapted experimental data from Demion et al., 2007 [89].

The TRPM4 channel’s current behavior is modeled and simulated based on Equations (8)–(10), following the principles of the Hodgkin–Huxley (HH) formalism, which incorporates activation and inactivation parameters. The equilibrium potential of TRPM4, denoted as E_Na_, is set at −40 mV. Activation parameters are computed across varying intracellular Ca^2+^ concentrations (Cai), with a baseline Cai concentration of 0.1 mM during rest. In Figure 2, we present the steady-state activation curves for the TRPM4 channel, comparing our model’s simulated curve (depicted by the solid red line) with experimental data (shown as filled triangles) sourced from Demion et al. (2007) [89]. The steady-state activation curve is unitless and ranges between 0 and 1. The x–axis is the logarithmic value of the Cai value. The comparison demonstrates a close alignment between our simulated curve and the experimental data. Notably, higher concentrations of Cai correspond to increased steady-state activation values, indicating an augmented TRPM4 current.

Subsequently, we integrated the TRPM4 channel into a single-compartment DSM model, as outlined by [83]. Earlier sections of this paper have detailed the inclusion of various ion channels within the DSM model. Before introducing the TRPM4 channel, we meticulously evaluated the stability, robustness, and flexibility of the DSM cell model, adhering to the methodologies outlined in the Section 4. Minor adjustments were made to certain parameters (particularly to ion channel conductances and time constant values) to ensure the stability of the resting membrane potential. The resting intracellular calcium concentration was maintained at 150 nM. Table 1 provides a comprehensive list of all ion channels incorporated into the model, along with their respective maximum conductances necessary for maintaining the stable resting membrane potential.

**Figure 3:**
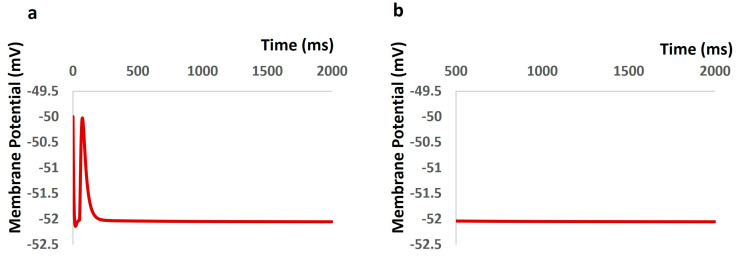
DSM model showing initial fluctuation (**a**) and constant resting membrane potential maintained at −52 mV (**b**).

By incorporating all ion channel models, including TRPM4, our primary aim was to uphold a physiological resting membrane potential (RMP) of −52 mV. The model’s robustness was confirmed by maintaining the RMP at −52 mV for 2000 ms, as illustrated in Figure 3. However, at the outset (0 ms), the model experienced a brief period of instability due to the behavior of all ion channels, resulting in slight fluctuations in membrane potential (Figure 3a). To address this, the time scale (x–axis) in Figure 3b begins from 500 ms onwards, excluding these initial fluctuations.

**Figure 4:**
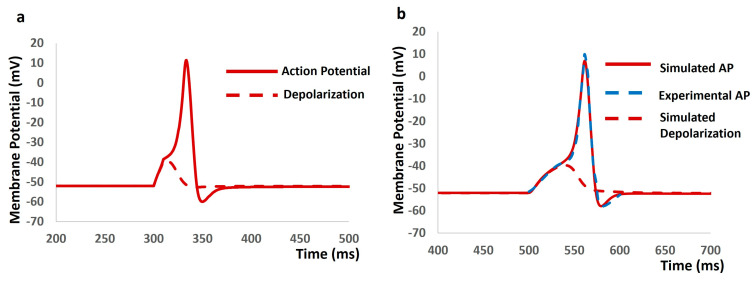
(**a**) The model generated AP (solid red line) and depolarization (dashed red line) with the current stimulus. (**b**) The model generated AP (solid red line), experimental AP (solid blue line), and simulated depolarization (dashed red line) with synaptic input stimulus.

We administered current stimuli with varying amplitudes lasting 10 ms to investigate evoked depolarization, action potential (AP) generation, and threshold estimation. No spikes occurred until the stimulus reached 0.56 mA, at which point the AP was triggered (Figure 4a). Analysis of the AP (depicted by the solid red line) and depolarization (represented by the dashed red line) facilitated the prediction of the threshold required to initiate the AP, estimated at −38.56 mV. Subsequently, we introduced synaptic input with different amplitudes to study evoked depolarization, AP generation, and threshold prediction. No spikes were observed with a stimulus of 0.0078 µS, while an AP was elicited with a stimulus of 0.0079 µS. Analysis of the simulated AP (depicted by the solid red line) and depolarization (shown as the dashed red line) predicted the threshold for AP initiation at −38.42 mV. Experimental data on AP in mice’s DSM cells under synaptic stimulus, as reported by [83], were compared with our model-simulated AP. The extracted data from this experimental AP (illustrated by the dashed blue line) exhibited a close match with our model’s output, corroborating the accuracy of our model.

**Figure 5:**
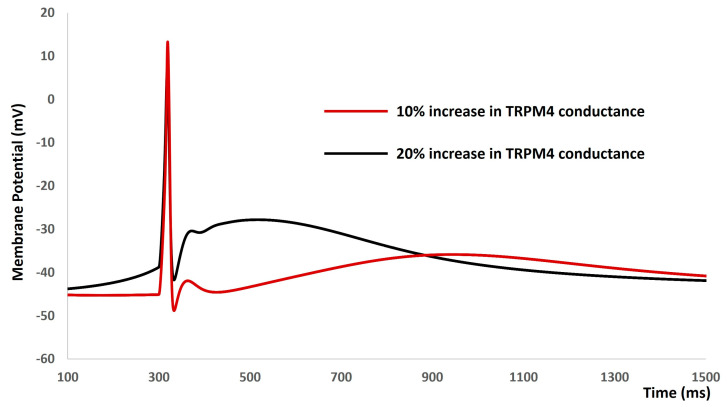
DSM action potential after a 10% (solid red line) and 20% (solid black line) increase in the maximum conductance of the TRPM4 ion channel.

Our subsequent investigation aimed to explore the modulatory impact of the TRPM4 channel on the electrical characteristics of the DSM cell. It is important to highlight one of the most intriguing aspects of DSM cell action potential generation, namely the variability in action potential shapes. Unlike neuronal and cardiac cells, which typically exhibit uniform action potentials for a given cell type, isolated DSM cells can produce various types of action potentials. These variations stem from differences in action potential duration, the shape of depolarizing and repolarizing phases, after-depolarization potential, and after-hyperpolarizations. Figure 5 depicts one type of DSM action potential following a 10% and 20% increase in the maximum conductance of the TRPM4 ion channel. The action potential represented by the solid red line arises after a 10% increase in the maximum conductance of the TRPM4 ion channel. Notably, the resting membrane potential becomes more positive following action potential generation. However, this generated action potential aligns with physiological expectations. In contrast, the action potential depicted by the solid black line emerges after a 20% increase in the maximum conductance of the TRPM4 ion channel. Although an action potential is generated, this particular type of action potential does not match physiological norms. Elevated conductance of the TRPM4 channel induces model instability.

**Figure 6:**
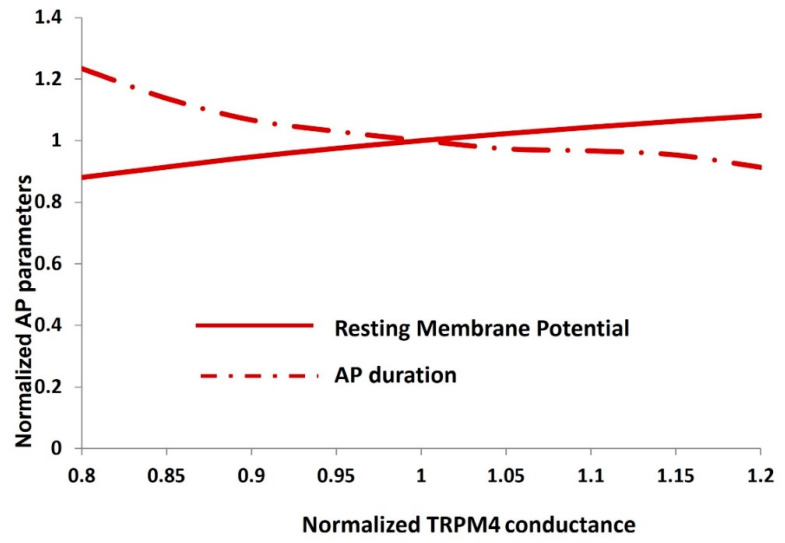
Sensitivity analysis of the TRPM4 channel conductance for DSM resting membrane potential and action potential duration.

To conduct a more comprehensive quantitative examination, we performed sensitivity analysis on the TRPM4 conductance concerning action potential parameters. Figure 6 illustrates the alterations in action potential parameters, specifically the resting membrane potential (solid red line) and action potential duration (dashed–dotted red line), in response to variations in the maximum conductance of the TRPM4 channel. All parameters were normalized for enhanced clarity. It is noteworthy that each action potential parameter fluctuates within a range of up to +20% and −30%, indicating the stability of the action potential across the range of TRPM4 channel conductance.

**Figure 7:**
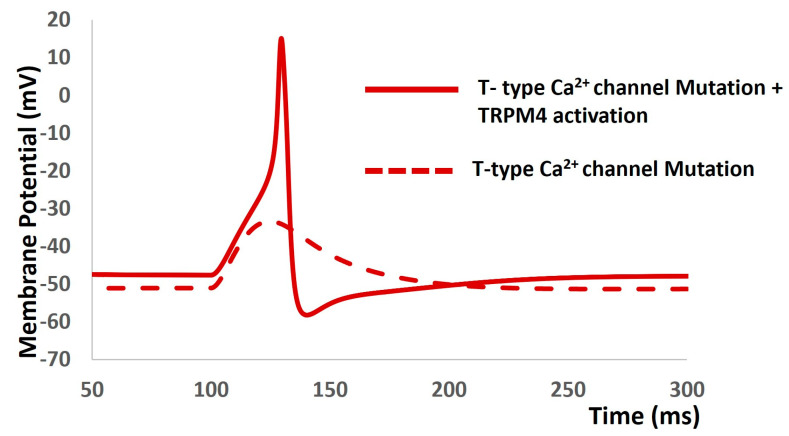
DSM cell generates action potential (solid red line) with mutation of T-type Ca^2+^ channel. The dashed line depicts the absence of action potential.

The prevalence of ion channels in excitable cells (such as neurons, cardiac cells, and muscle cells) prompts fundamental inquiries into how the unique intrinsic characteristics of individual cells arise from their specific complement of channels. Across all excitable cells, many ion channels exhibit overlapping voltage and time-dependent traits. We propose that these shared properties contribute to the robustness of physiological function. Despite displaying consistent and similar behaviors, individual excitable cells of the same type demonstrate variability in ion channel conductance densities [61,90]. This complexity complicates the direct function assignment to any particular conductance, and is associated with the diverse responses of similar cells to disturbances, deletions, and pharmacological treatments. Several experimental investigations have shown that the activation of the T-type Ca^2+^ channel depolarizes the DSM cell to the threshold of the L-type Ca^2+^ channel, for which the cell generates AP [91]. To examine our hypothesis concerning a mutation in the T-type Ca^2+^ channel (whereby the maximum conductance of the T-type Ca^2+^ channel is set to zero), we adjusted the conductance of the TRPM4 channel to maintain the electrophysiological behavior of the DSM cell. As illustrated in Figure 7, when the T-type Ca^2+^ channel was muted (depicted by the dashed red line), the DSM cell failed to generate any action potential. Further adjustments to the parameters of the TRPM4 channel rendered the DSM cell incapable of generating action potentials. However, by fine-tuning the parameters of the TRPM4, L-type Ca^2+^ channel, and inward rectifying channel, the DSM cell was able to generate action potentials without the presence of the T-type Ca^2+^ channel (depicted by the solid red line). Initially, the model exhibited stochastic behaviors, but subsequent adjustments to other ion channel parameters enabled the generation of action potentials. This finding strongly supports the variability and resilience of our computational model. It should be noted that other ion channel blockers were applied in the DSM models, and their effects on AP were shown in [83].

## 3. Discussion

OAB syndrome has a profound impact on people’s lives, and poses significant financial challenges. This condition, characterized by sudden and uncontrollable urges to urinate, often results in frequent bathroom visits, disrupted sleep, and limitations in daily activities. The anxiety and embarrassment surrounding potential accidents can lead to social isolation and reduced productivity. Additionally, managing OAB requires ongoing medical expenses, such as doctor visits, medication, and possibly surgery, which places strain on both personal finances and healthcare systems. Despite its widespread occurrence and consequences, much remains unknown about the underlying causes and effective treatments for OAB. Fundamental research plays a crucial role in unraveling the complexities of this condition, including understanding the neural pathways involved in bladder control, identifying early detection biomarkers, and developing targeted therapies with minimal side effects. DSM contraction is closely linked to OAB syndrome [16,17], as heightened activity in these muscles can lead to involuntary and frequent bladder contractions, resulting in the characteristic urgency and urge incontinence experienced by individuals with OAB. A thorough exploration of the underlying biophysical mechanisms behind DSM electrophysiology is crucial for identifying new pharmacological targets with minimal side effects. As a mechanical event, the contraction is initiated by intracellular calcium elevation and the generation of action potential. Ion channels play a crucial role in modulating the underlying electrical properties, including the action potential, across the DSM cell membrane. Hence, accurately quantifying the individual ion channel’s contribution to modulating the action potential will aid the exploration of more effective drugs for overactive bladder treatment. In recent years, multiple experimental findings have indicated the presence of the TRPM4 ion channel in DSM cells and its role in modulating DSM contraction [49,50]. Due to the highly complex nature of experimental smooth muscle electrophysiology setups, the quantified contribution of the TRPM4 channel to DSM cell membrane potential and action potential has not yet been studied.

Computational and in silico approaches have revolutionized the field of electrophysiology by providing valuable insights into the behavior of ion channels at a fundamental level. These methods simulate the complex interactions of ion channels with high precision, allowing researchers to explore diverse scenarios and predict ion channel behavior under different conditions. By complementing experimental studies, computational models enhance our understanding of ion channel function, kinetics, and pharmacology. Moreover, they offer a platform for hypothesis testing and guide experimental design, ultimately accelerating the discovery of novel targets and therapies for various physiological and pathological conditions. While numerous computational models have been published regarding DSM cell electrophysiology, none of them has incorporated the TRPM4 channel for investigation [82,83,84,86,87]. Computational modeling is an ongoing process, wherein established models are continuously refined, with new experimental findings yielding more fruitful research outcomes. To the best of our knowledge, the model presented by us is the first biophysically constrained DSM cell model to investigate the contribution of the TRPM4 channel to DSM cell excitability. 

Our first objective in developing this model was to employ differential equations, Hodgkin–Huxley formalisms, and parameters to accurately replicate the internal kinetics of the TRPM4 channel. We have depicted the simulated steady-state activation curve across various calcium concentrations (Figure 2). Validation against experimental data reinforces the heightened accuracy of our TRPM4 ion channel model, confirming the successful achievement of our first objective. Our second objective was to integrate the TRPM4 channel into a DSM cell model and examine its modulating effects on cellular excitability. Before integrating the new TRPM4 channel, we validated the previous model to ensure its robustness and reproducibility. Once we confirmed the proper functioning of the model, we proceeded with the integration of the TRPM4 channel. However, adding a foreign element to an established model often results in aberrant stochastic behavior. Similarly, our model exhibited instability after the incorporation of the TRPM4 channel. To address this, we carefully adjusted the maximum conductances of ion channels to stabilize the model and replicate previously simulated outputs. We validated the simulated synaptic potentials and action potentials by comparing them with experimental data, confirming our model’s ability to replicate experimental findings [83] (Figure 4). Subsequently, we adjusted the maximum conductance of the TRPM4 channel within physiological ranges to assess its impact on the resting membrane potential, action potential threshold, and peak amplitude. Activation of the TRPM4 channel leads to a shift in the resting membrane potential towards a more positive state, reduces the threshold potential for action potential initiation, and increases the peak amplitude of the action potential. This suggests that overexpression of TRPM4 channels across the DSM cell membrane could induce heightened spontaneous contractions, a primary symptom of overactive bladder [49,50]. Therefore, controlled doses of TRPM4 channel blockers may mitigate this pathological condition. Conversely, in absence of overexpressed channels, their inherent activity may still lead to overactivity due to spontaneous intracellular calcium elevation. Experimental studies have demonstrated transient increases in intracellular calcium, such as calcium sparks, puffs, and waves [92], which could activate TRPM4 channels locally, exacerbating overactive bladder symptoms. The term “localization” distinguishes TRPM4 channel activation from calcium-dependent potassium channel activation, which hyperpolarizes the membrane, reducing cellular excitability. To further validate our model, we conducted a sensitivity analysis, providing additional quantitative insights into how the TRPM4 channel influences cellular electrical properties. As a primary goal of any computational physiology model is to generate new hypotheses, our third objective was to propose novel insights into the TRPM4 channel in detrusor muscle biophysics. In the introduction, we briefly discussed the redundancy of ion channel types to ensure resilience against perturbations. Building on this concept, we inactivated the T-type calcium channel by reducing its maximum conductance to zero, then simulated all electrical properties by adjusting conductances and other parameters for the remaining ion channels. This revealed that the TRPM4 channel could compensate for the absence of the T-type calcium channel. Our hypothesis suggests that the TRPM4 channel may serve as a standby ion channel to maintain bladder physiology in cases of dysfunction or mutation in the T-type calcium channel.

Computational models inherently have several limitations. Firstly, our current model investigated the modulating effects of TRPM4 channels on spike-type action potentials in the DSM model. However, DSM cells also generate pacemaking-type action potentials, which are not simulated in this model. To date, no biophysically detailed DSM model has been developed to simulate pacemaking action potentials. Additionally, most of the experimental research papers highlighted the spike-type action potentials in single isolated DSM cells [82,83]. Although the action potential was validated against experimental data from mice’s urinary bladders, our ion channel parameters were derived from other animals (mice and guinea pigs) and organs, due to a lack of specific data [82,83]. Additionally, while actual smooth muscle morphology may not perfectly align with a cylindrical shape, our model is designed based on this simplified geometry. Furthermore, DSM cells function as a syncytium, where multiple cells are interconnected via gap junctions, enabling electrical signal propagation. However, our model is limited to a single-compartment isolated cell. Future model expansions could involve investigating the effects of TRPM4 channel modulation in multicompartmental tissues with complex calcium dynamics.

## 4. Materials and Methods

### 4.1. Model Adaptation

Biophysically detailed cell modeling for neuronal cells made its debut in 1952 in neuroscience, thanks to the groundbreaking work of Hodgkin and Huxley on the squid giant axon [93]. In the 1960s, Noble pioneered the first models of cardiac cellular activity [94]. Since then, an impressive array of mathematical models for neuronal cells, cardiac muscle, and smooth muscle electrophysiology have emerged. The complexity of these models has steadily increased over the years as more experimental data have become available. Several DSM cell electrophysiology models have been mathematically designed to mimic the behavior of DSM electrical activities [82,83,84,86,87]. These models also serve as a bridge between cellular-level models and tissue-level function, offering a comprehensive understanding of DSM cell electrical activity across different scales. For establishing a single-compartment DSM AP model to integrate the TRPM4 ion channel, we have adapted the model [83] for simulating DSM cell electrical properties. The DSM cell membrane’s cylindrical single-cell morphology and passive electrical properties are derived from the experimental data for a single isolated DSM cell [95]. The length, diameter, membrane resistivity, cytoplasmic resistivity, and membrane capacitance are 200 μm, 6 μm, 138 kΩ.cm^2^, 183 Ω.cm, and 1 μF/cm^2^ respectively. 

### 4.2. General Membrane Current Descriptions

**Figure 8:**
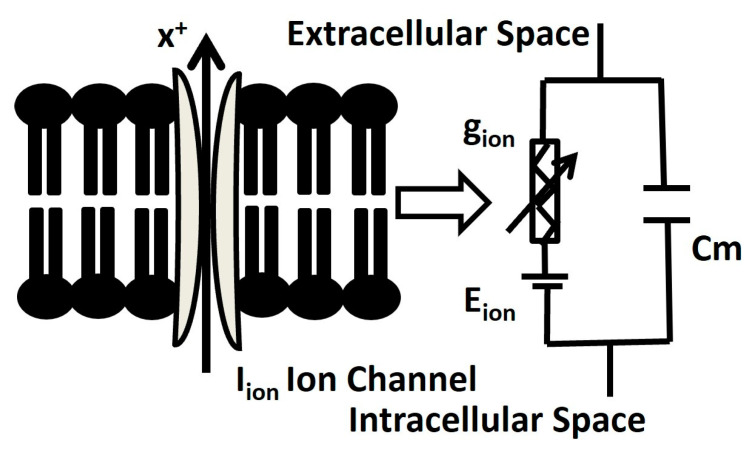
Schematic overview of parallel conductance model for ionic current. Further elucidation is provided in the subsequent paragraph.

The DSM cell is electrically defined by a resistor–capacitor (RC) circuit, where the membrane capacitance C_m_ is parallel with the variable ion channel conductance g_ion_. All active ion channel conductances g_ion_ are associated with respective Nernst potential E_ion_ in series. Figure 8 illustrates the schematic overview of the parallel conductance model for ionic current (I_ion_), and it shows the flow of ion ‘X^+^’ under the influence of an electrochemical driving force. 

The calculation of the individual ionic current follows the principles of Ohm’s law, traditionally expressed through Equation (1).

(1) $$I_{ion}=\bar{g}{\left[m(V_{m},t,{\left[{Ca}^{2+}\right]}_{i})\right]}^{x}{\left[h(V_{m},t,{\left[{Ca}^{2+}\right]}_{i})\right]}^{y}(V_{m}-E_{Nernst})$$

In Equation (1), $\bar{g}$ and E_Nernst_ represent the maximum channel conductance and Nernst potential specific to the ion channel under consideration. The variable parameters m and h correspond to dimensionless activation- and inactivation-gating variables, respectively, which are dependent on time, voltage, and Ca^2+^ concentration. To facilitate equation fitting, an additional pair of dimensionless parameters denoted as ‘x’ and ‘y’ are introduced, determined through a system of first-order differential equations, following the classical Hodgkin–Huxley (HH) formulation mechanisms [93].

For example, Equation (2) computes the instantaneous value of the activation variable “m” in our DSM cell model.

(2) $$\frac{\;dm(V_{m},\;t)}{dt}=\frac{m_{\infty \;}(V_{m})-m(V_{m},t)}{\tau_{m}}$$

In this equation, m_∞_ represents the steady-state value, and τ_m_ denotes the time constant, both of which are functions of voltage and/or ionic concentrations.

In this context, the relationship between the state parameter and the membrane potential (V_m_) for ion channels is elucidated through the Boltzmann equation.

(3) m∞=11+exp⁡((Vm+Vm12)/Sm)

where V_1/2_ is the half activation potential and S is the slope factor. 

**Figure 9:**
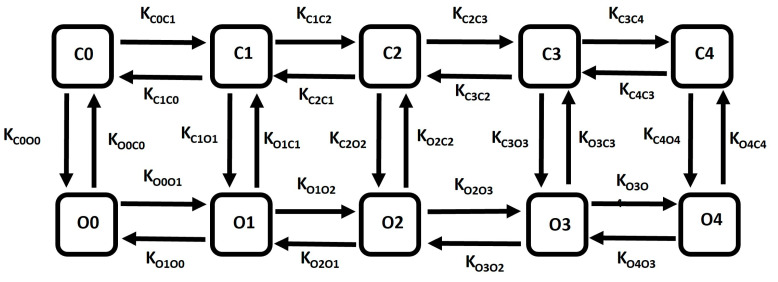
Schematic diagram of 10-state Markov model for BK channel. A detailed explanation is provided in the text.

The large conductance calcium-activated potassium (BK) channel kinetics have been elucidated through a multi-state Markov model (MM) [96], which offers a detailed representation of the channel’s sensitivity to Ca^2+^, enhancing precision. Figure 9 illustrates the schematic diagram of the 10-state Markov model for the BK channel. The MM model enhances the accuracy of the description of a multi-agent activated ion channel by incorporating multiple closed and open states to represent its internal conditions. This model comprises closed states denoted as C0, C1, C2, C3, and C4, and five corresponding open states denoted as O0, O1, O2, O3, and O4. Among these, the open state O4 facilitates the passage of K^+^ ions via BK channels driven by the instantaneous electrochemical driving force (EDF). The BK current (I_BK_) is determined using the following equation:

(4) $$I_{BK}=\bar{g_{BK}}\times O\times (V-E_{K})$$

where $\bar{g_{BK}}$ is the maximum conductance and O is the summation of O1, O2, O3, and O4. 

Common rate equations:

K_on_ = 335, K_coff_ = 26, K_ooff_ = 26, O = O1 + O2 + O3 + O4 (5)

Rate equations for voltage-dependent transitions:

K_C0O0_ = 0.03162 × a, K_C1O1_ = 0.000969 × a, K_C2O2_ = 0.0000381 × a, K_C3O3_ = 0.000881 × a, K_C4O4_ = 0.054324 × a, K_O0C0_
= 328.1084 × b, K_O1C1_ = 154.1736 × b, K_O2C2_ = 33.6594 × b, K_O3C3_= 0.097312 × b, K_O4C4_ = 0.000406 × b × cai (6)

State equations for calcium (cai)-dependent transitions:

K_C0C1_ = 3 × K_on_ × cai, K_C1C2_ = 4 × K_on_ × cai, K_C2C3_ = 3 × K_on_ × cai, K_C3C4_ = K_on_ × cai K_C4C3_ = 3 × K_coff_ × cai, K_C3C2_ = 4 × K_coff_ × cai, K_C2C1_ = 3 × K_coff_ × cai, K_C1C0_ = K_coff_ × cai K_O0O1_ = 3 × K_on_ × cai, K_O1O2_ = 4 × K_on_ × cai, K_O2O3_ = 3 × K_on_ × cai, K_O3O4_ = K_on_ × cai K_O4O3_ = 3 × K_ooff_ × cai, K_O3O2_ = 4 × K_ooff_ × cai, K_O2O1_ = 3 × K_ooff_ × cai, K_O1O0_ = K_ooff_ × cai (7)

In Equations (5)–(7), the parameter values are defined to obtain the most accurate model of the BK ion channel, aiming to simulate the current–voltage relationship curve and the BK current under different membrane potentials and internal Ca^2+^ concentration.

### 4.3. TRPM 4 Channel with Ca^2+^ Sensing Mechanism 

Here, the mathematical interpretation of the TRPM4 ion channel is based on conventional Hodgkin–Huxley formalism. In this model, Hodgkin–Huxley formalism is adapted for I_Na_ ionic currents in Equation (8):

(8) $$I=\bar{g}{zm}^{x}\left(V_{m}-E_{rev}\right)$$

where $\bar{g}$ is maximum conductance, E_rev_ is Nernst potential for sodium ion, m is intracellular calcium concentration-dependent activation variable, and x is the power for the gating variable.

The first-order differential equation is used in Equation (9) to describe the time and calcium-dependent nature of gating variable m:

(9) $$\frac{dm}{dt}=\frac{\left(m_{\infty }-m\right)}{\tau_{m}}$$

where m_∞_ and τ_m_ are defined as the steady-state value and time constant of gating variable ‘m’ respectively.

Here the activation steady state is expressed by the Hills equation:

(10) z∞=1(1+CahCain)

where Cah is half inhibition intracellular calcium ion centration, Cai is free calcium concentration, and n is the Hill coefficient. All modeling parameters are borrowed from literature based on experimental studies. Cah and n are fitted to match with the experimental result. 

### 4.4. Whole DSM Cell Model and Simulation

**Figure 10:**
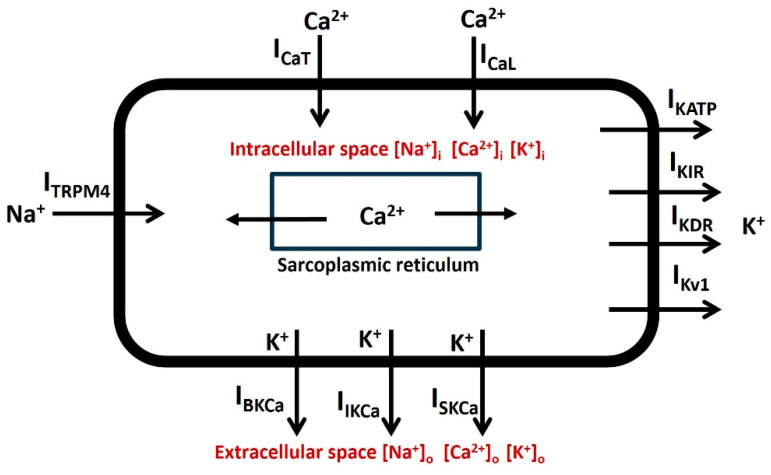
Schematic diagram illustrating all ionic components within a DSM cell. The accompanying paragraph provides descriptions for each component.

Figure 10 shows the list of active ion channels incorporated into our DSM cell. In our model, the two types of voltage-gated Ca^2+^ channels are T-type (I_CaT_) and L-type (I_CaL_) Ca^2+^ channels. Voltage-gated *K*^+^ channels are inward-rectifying channels (I_KIR_), delayed rectifier channels (I_KDR_), and fast (I_Kv1_) channels. Ca^2+^-activated *K*^+^ channels are large conductance (I_BKCa_), intermediate conductance (I_IKCa_), and small conductance (I_SKCa_) channels. The TRPM4 channel (I_TRPM4_) and leakage channels (I_l_) have also been added to this single-compartment DSM cell model. 

The synaptic input defined by the alpha function [97] or a brief square pulse of injected current (variable duration and magnitude) serves as the external stimulus to generate action potentials in the whole-cell model. Subsequently, the conductance is adjusted to explore the modified response in action potentials and resting membrane potential. Simulations were conducted on a PC equipped with an Intel Core i7 CPU operating at 3.80 GHz and with a dual-core setup. The NEURON simulation environment was utilized for model creation, renowned for its accurate portrayal of excitable cells [98]. While NEURON primarily employs implicit integration techniques, like backward Euler and a variant of Crank-Nicolson for stability, it also supports Euler’s method due to its efficiency and minimal memory usage [99]. In NEURON, the actual simulation time, t, is measured in milliseconds, and at each time step it is incremented by dt. A smaller dt is required for more accurate results. The simulation speed depends on the complexity and modeling approaches used for the network. To balance simulation speed and accuracy, we set dt to 0.04 ms. We also adapted NEURON’s multiple run fitter optimization algorithms to optimize our running fitness for action potential generation. This process usually entails scrutinizing different aspects of our running performance and leveraging the gathered data to refine our training routine, aiming for improved outcomes.

Following model creation, stability and consistency were evaluated by adjusting the maximum conductance (gmax) of ionic conductances within ±30% of the default value. Results demonstrated stable action potentials exhibiting anticipated responses to changes in conductance; for instance, increased gmax led to higher AP peak amplitudes while preserving the AP’s normal characteristics. The experimental data used for validation in our current model are borrowed from a previously published paper from 2018 [83], which involved a collaboration for obtaining experimental data recordings. Goodness-of-fit (GoF) involves assessing how well the observed data align with the data predicted by a model, typically through a fit statistic or measure of deviation, such as residuals, Chi-square, or deviance. The standard error of the regression (S) or root mean squared error (RMSE) serves as a measure of GoF in our analyses of simulated action potentials, chosen due to the inadequacy of R^2^ for nonlinear comparisons [100]. S is computed using the following formula:

(11) $$S=\sqrt{\frac{\Sigma {\left(Y_{Exp}-Y_{Sim}\right)}^{2}}{M-N}}$$

In Equation (11), Y_expt_ represents the experimental value, Y_Sim_ denotes the corresponding simulated fit value, N stands for the number of parameters utilized in the fitting equation, and M represents the total number of data points. A smaller S value indicates lower average errors and indicates a better fit. We established a threshold for a satisfactory model as 5% of the difference between the maximum and minimum values observed in the experimental data. Any S value below this threshold is deemed indicative of a good fit.

The model code will be accessible on GitHub and the repository (https://modeldb.science, accessed on 5 May 2024) for open-source code sharing.

## Figures and Tables

**Table 1:** Maximum conductances of ion channels.

Ion Channel	Conductance (S/cm^2^)
T-type Ca^2+^ channel	0.0002
L-type Ca^2+^ channel	0.0003
Voltage-gated K^+^ channel-Kv1	0.0006
Voltage-gated K^+^ channel-KDR	0.0009
Calcium-dependent K^+^ channel (BK)	0.0008
Calcium-dependent K^+^ channel (IK)	0.0007
Calcium-dependent K^+^ channel (SK)	0.0001
ATP-dependent K^+^ channel	0.0001
Inward-rectifying channel	0.0001
TRPM4 Channel	0.0002

## Data Availability

Data are contained within the article.
